# A Hybrid Active Neutral Point Clamped Inverter Utilizing Si and Ga_2_O_3_ Semiconductors: Modelling and Performance Analysis

**DOI:** 10.3390/mi12121466

**Published:** 2021-11-27

**Authors:** Sheikh Tanzim Meraj, Nor Zaihar Yahaya, Molla Shahadat Hossain Lipu, Jahedul Islam, Law Kah Haw, Kamrul Hasan, Md. Sazal Miah, Shaheer Ansari, Aini Hussain

**Affiliations:** 1 Department of Electrical and Electronic Engineering, Universiti Teknologi PETRONAS, Seri Iskandar 32610, Perak, Malaysia; sheikh_19001724@utp.edu.my (S.T.M.); norzaihar_yahaya@utp.edu.my (N.Z.Y.); 2 Department of Electrical, Electronic and Systems Engineering, Universiti Kebangsaan Malaysia, Bangi 43600, Selangor, Malaysia; p100855@siswa.ukm.edu.my (S.A.); draini@ukm.edu.my (A.H.); 3 Centre for Automotive Research (CAR), Universiti Kebangsaan Malaysia, Bangi 43600, Selangor, Malaysia; 4 Department of Fundamental and Applied Sciences, Universiti Teknologi PETRONAS, Seri Iskandar 32610, Perak, Malaysia; jahedul_17010697@utp.edu.my; 5 Faculty of Engineering, Universiti Teknologi Brunei, Bandar Seri Begawan 1410, Brunei; kahhaw.law@utb.edu.bn; 6 School of Electrical Engineering, College of Engineering Studies, Universiti Teknologi MARA, Shah Alam 40450, Selangor, Malaysia; 2019984679@isiswa.uitm.edu.my; 7 School of Engineering and Technology, Asian Institute of Technology, Pathumthani 12120, Thailand; st121577@ait.asia

**Keywords:** power electronics, ultrawide bandgap, semiconductors, neutral point clamped, inverter, silicon, gallium trioxide, fabrication, hybridization

## Abstract

In this paper, the performance of an active neutral point clamped (ANPC) inverter is evaluated, which is developed utilizing both silicon (Si) and gallium trioxide (Ga_2_O_3_) devices. The hybridization of semiconductor devices is performed since the production volume and fabrication of ultra-wide bandgap (UWBG) semiconductors are still in the early-stage, and they are highly expensive. In the proposed ANPC topology, the Si devices are operated at a low switching frequency, while the Ga_2_O_3_ switches are operated at a higher switching frequency. The proposed ANPC mitigates the fault current in the switching devices which are prevalent in conventional ANPCs. The proposed ANPC is developed by applying a specified modulation technique and an intelligent switching arrangement, which has further improved its performance by optimizing the loss distribution among the Si/Ga_2_O_3_ devices and thus effectively increases the overall efficiency of the inverter. It profoundly reduces the common mode current stress on the switches and thus generates a lower common-mode voltage on the output. It can also operate at a broad range of power factors. The paper extensively analyzed the switching performance of UWBG semiconductor (Ga_2_O_3_) devices using double pulse testing (DPT) and proper simulation results. The proposed inverter reduced the fault current to 52 A and achieved a maximum efficiency of 99.1%.

## 1. Introduction

Silicon-based devices have primarily been used and are still dominant in developing power inverters [1,2]. However, ultra-wide bandgap (UWBG) semiconductors have gained a significant amount of attention in recent years [3]. As a result, Ga_2_O_3_ being a strong candidate for UWBG devices have the potential to be profoundly applied in the various applications in the field of power electronics ranging from Photovoltaic (PV) inverters and UPS systems to inverters for traction and space applications, among others [4,5,6]. In these power inverters, UWBG semiconductors can contribute to high efficiency, inverter size reduction, and high-temperature environment operation, which are unlikely to be achieved otherwise [7]. These features of Ga_2_O_3_ devices are due to the specific properties of the UWBG material. Gallium trioxide (Ga_2_O_3_) devices are capable of achieving these because unlike their conventional counterpart, the blocking voltage that is rated for these devices is nearly a hundred times higher for the same width of the drift region [8]. In addition, the high thermal conductivity, along with the fast-switching speed are two main factors that are offered by Ga_2_O_3_ devices to gain this advantage [3]. Though the UWBG devices can be applied in medium power applications, the ongoing research has suggested that these devices have great potential to be applied in high power applications with modular multilevel inverters (MLIs) [9].

For medium-voltage-range Photovoltaics (PV), several DC-link voltages are proposed in recent years [10,11] considering different interests. However, when efficiency and reliability are the main concern, the 1.5kV DC-link-based PV generation system has gained significant attention along with systems [12,13]. In addition to that, for higher efficiency in PV systems, transformer-less configurations have shown better performance compared to other transformer-based configurations [6]. Though many inverter topologies had been proposed previously considering high voltage applications, a three-level neutral point clamped (NPC) inverter is one of the most optimal inverter choices for high voltage applications [14]. Since the clamped common-mode voltage (CMV) is enabled in this type of topology, it minimizes leakage-current-related issues [15]. That is why, for a transformer-less system, it is a better choice than other systems which are incorporated by leakage current. Despite this, due to the unequal loss distribution among the switches, the NPC inverter has issues related to neutral-point voltage imbalance, as well as a shoot-through fault in the switching devices [16].

Various types of control strategies along with modified inverter topologies have recently been proposed to overcome the inconveniences in the NPC inverter. Since NPC inverters are prone to the shoot-through problem, a split inductor configuration can be used to solve this issue [17]. It should also be noted that in addition to successfully protecting the shoot-through fault, reduced leakage current and the eradication of CMV transitions that are high frequency in nature, can be achieved through this inverter. However, even though this configuration removes most of the inconveniences, it operates in a unity power factor region. Therefore, this configuration is hardly suitable for high voltage applications that are designed specifically for supplying reactive power to the grid. In addition to that, the previously mentioned non-uniform loss distribution problem in the switches of the NPC inverters still exists in these topologies. 

In [18], the non-uniform current distribution was addressed, and it proposed an active neutral point clamped (ANPC) inverter. As per the switching states of ANPC inverters, additional redundant zero states can be gained in the ANPC inverter topology. Therefore, unequal switching loss distribution can be mitigated if different zero states can be appropriately exploited in the switching states of the ANPC inverter. Considering these additional states, some notable PWM-based control techniques are employed previously in the ANPC inverter topology [19,20]. The use of current and voltage sensors for the selection of the redundant zero states that are available in the ANPC topology focused on power factors. So, how the states of the inverters will be chosen is largely related to the feedback signals of those current and voltage sensors. This solution is optimized to achieve high efficiency in ANPC, which provides the states for the hybrid Si/Ga_2_O_3_-devices-based ANPC topology. The high efficiency and the low cost relative to all Ga_2_O_3_ inverters can be obtained according to researchers [21]. Recent literature has also shown promising results using the aforementioned approach where the switching devices of the ANPC were mostly built using wide bandgap (WBG) materials or silicon carbide (SiC) [22]. However, one fact about their research is that they only considered low voltage applications, and the entire ANPC inverter was built using devices from the same bandgap materials. Furthermore, one fact about their research is that they considered the converters suitable for only low-power applications having low voltages. Because in the case of MV applications, unlike silicon devices, the body diodes of Ga_2_O_3_ MOSFETs are the cause of further switching losses along with overshoots that are significant in switching transient, the design criteria would be different [23]. In addition to that, as the dead-band time is declined in high-frequency devices, the severity of the shoot-through fault rises remarkably. It should also be noted that as high-frequency switching devices are employed at the output side, an increase has been seen in the voltage amplitude in the electromagnetic interference (EMI) frequency range, which ultimately contributes to the increased size and complexity in EMI filters [24].

Considering the issues stated above, this study proposes a hybrid ANPC inverter that utilizes both conventional Si and Ga_2_O_3_ devices. As a result of this hybridization, the switching losses of the inverter are reduced significantly. The hybridization also made the implementation of a split-output structure achievable. Thus, the proposed circuit can also handle the switching transient overshoots. In this structure, since the UWBG switch is decoupled externally by the parallel diode, both overshoot issues in the switching transient, as well as switching losses, are declined significantly. These reduced overshoots ultimately also lead to decreased voltage and current stresses on the UWBG devices. As this converter topology is capable of supplying reactive power to loads with a wide range of power factors, it can be used for grid-tied PV systems. The key contributions of the paper can be listed as follows:

- Incorporating UWBG semiconductors to utilize their various advantages such as reduced size, minimized switching transient overshoots, reduced current and voltage stress, high-frequency switching, and efficiency;
- Hybridization with conventional Si switches to prevent high leakage current and high-frequency switching losses;
- The split-output structure is adopted for the ANPC inverter to prevent shoot-through current fault, reduce electromagnetic interference (EMI) on the output, and enable it to operate under different ranges of power factors;
- Validating the performance enhancement by comparing with conventional ANPC in terms of power losses, efficiency, fault current, and EMI.

The rest of the paper is arranged as follows. The modeling of the proposed inverter topology is outlined in Section 2. Following this section, a characteristic and comparative analysis of the proposed inverter and conventional ANPC is presented in Section 3, including an analysis on fault currents, core losses, switching losses, efficiencies, EMI, and power factors. Section 4 discusses the summary and conclusion of the manuscript.

## 2. Modelling of Hybrid ANPC Inverter with Ga_2_O_3_ and Si Switches

### 2.1. Modelling of UWBG (Ga_2_O_3_) Semiconductors

**Figure 1:**
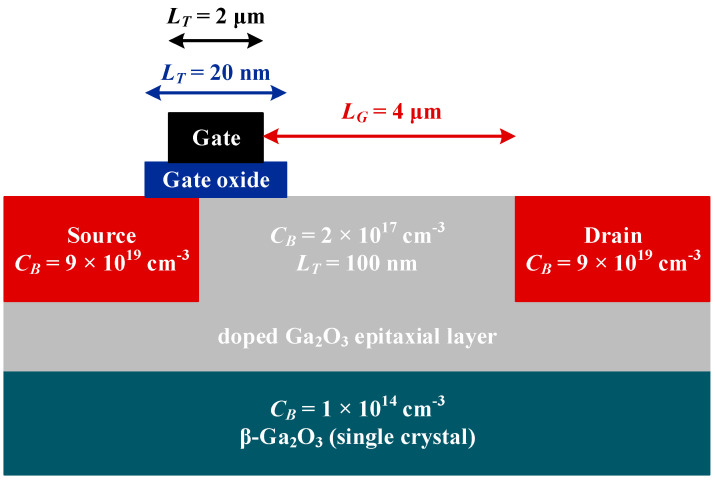
Modelling UWBG semiconductor switches.

**Figure 2:**
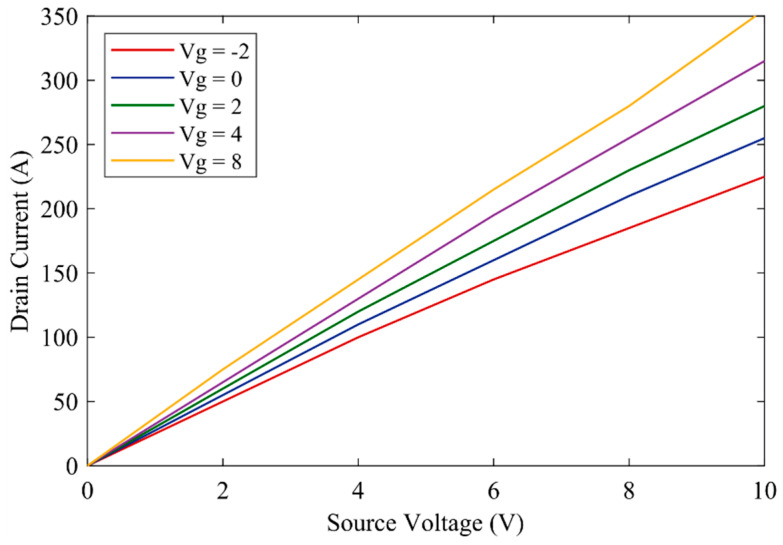
I-V characteristics of Ga_2_O_3_ semiconductor switches.

In this article, the design of UWBG semiconductors is described briefly since the modeling and fabrication of the UWBG semiconductor is not the main objective of this study. The UWBG switches are modeled considering the drain current and source implementation [25], while the channel is isolated using the doping structure as shown in Figure 1. The Ga_2_O_3_ parameters that are used in this study to build the proposed inverter are demonstrated in Table 1. These parameters are only used in technology computer-aided design (TCAD) to evaluate the conduction behavior of the Ga_2_O_3_ devices. The I-V characteristics of these switches are shown in Figure 2.

Firstly, a Ga_2_O_3_ n-type epitaxial layer having 100 nm thickness is developed over a β-Ga_2_O_3_ (single crystal), which is semi-insulating in nature. Secondly, A dopant with a concentration of 2 × 10^17^ cm^−3^ is applied to dope the epitaxial layer. Tin (Si/Sn) implantation is used to form the 50 nm deep drain regions and the dopant concentration. Finally, a metal gate of 2 μm length and a work function of 5.93 eV is implanted on the top of a dielectric film gate with 20 nm length. The drain and gate are separated by a 4 μm gap [26].

To evaluate the performance of the Ga_2_O_3_ devices, accurate switching behavior is very crucial. However, since the switching behavior of the UWBG devices cannot be evaluated using TCAD, SPICE models of the Ga_2_O_3_ are required for further analysis [27]. In this regard, the level 1 Schichman–Hodges model parameters as shown in Table 2 are extracted from TCAD and were used to develop the SPICE model. The model parameters along with the switching, conduction, drain-source voltage, and drain current are implemented in LTSpice software to build a simulation model of the Ga_2_O_3_ switching device. The parameters that are used to build the LTSpice simulation model are shown in Table 2.

### 2.2. Modelling of Hybrid ANPC Inverter

**Figure 3:**
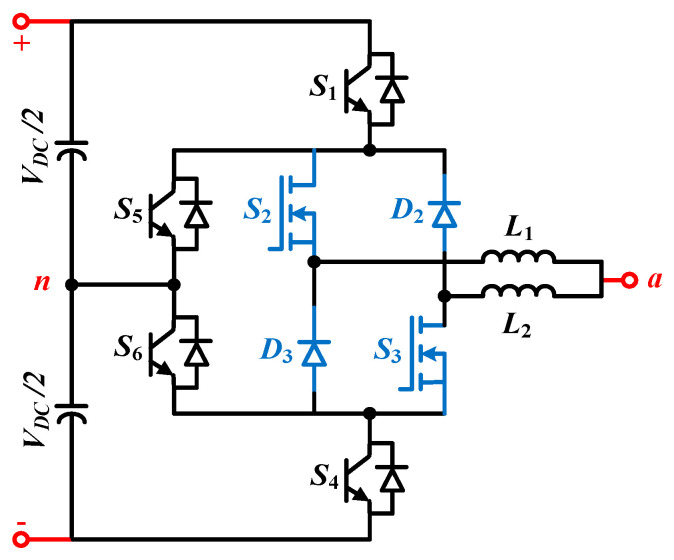
Schematic diagram of the hybrid ANPC inverter comprising UWBG switches.

**Figure 4:**
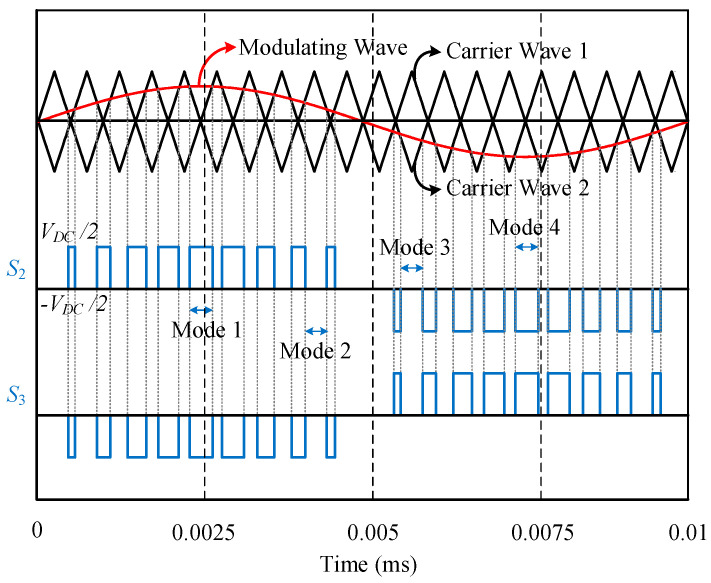
Switching pulses of the Ga_2_O_3_ devices of the hybrid ANPC using LSPWM.

The schematic diagram of the proposed topology is depicted in Figure 3. The four switches, namely, *S*_1_, *S*_4_, *S*_5_, and *S*_6_, are constructed by using Si-based IGBTs, which are rated as 1.2 kV. On the other hand, the *S*_2_ and *S_3_* switches are made by Ga_2_O_3_-based MOSFETs of 800 V rating. The utilization of both Si and Ga_2_O_3_ devices has ensured that the inductors can be split into *L*_1_ and *L*_2_ through these devices. In addition, it should be noted that the diodes *D*_2_ and *D*_3_ are both Ga_2_O_3_-based Schottky diodes [28]. As illustrated in Figure 3, the Ga_2_O_3_-based MOSFETs, i.e., *S*_2_ and *S*_3_ switches, are decoupled from *D_2_* and *D_3_*, and this leads to the division of the inductors. Some capacitors are series-connected in the DC-link to make up neutral point ‘*n*’. There is a common portion of the two inductors between point ‘*a*’ and the terminal ‘*n*’, and the output is taken from this portion. As it is listed in Table 1, this inverter has six possible states. The states denoted by *P* and *N* represent positive and negative states, respectively, and null states are referred to as *O*_1_ to *O*_4_. *S*_2_ and *S_3_* gallium trioxide (Ga_2_O_3_) switches are operated at a higher frequency, whereas Si IGBTs are operated in lower frequencies because it is required to maximize the output. To exploit this, only two null states, *O*_3_ and *O*_2_, as shown in Table 3, are utilized. More specifically, in case of the positive half cycle, the states *P* as well as *O*_3_ are used, and the states *N* and *O*_2_ are utilized for the operation of the negative half cycle. The UWBG is operated at a higher frequency of 100 kHz while the other four Si-based switches are operated at a lower fundamental frequency of 50 Hz. The gate pulses for switches are created using the level-shifted pulse width modulation (LSPWM) [29], which are depicted in Figure 4. The *S*_1_ and *S*_6_ switches will remain ON, while switches *S*_4_ and *S*_5_ will be turned OFF in case of positive cycle operation. On the contrary, the *S*_4_ and *S*_5_ switches will be ON and start conducting, while the *S*_1_ and *S*_6_ switches will be turned off for the negative half cycle.

**Figure 5:**
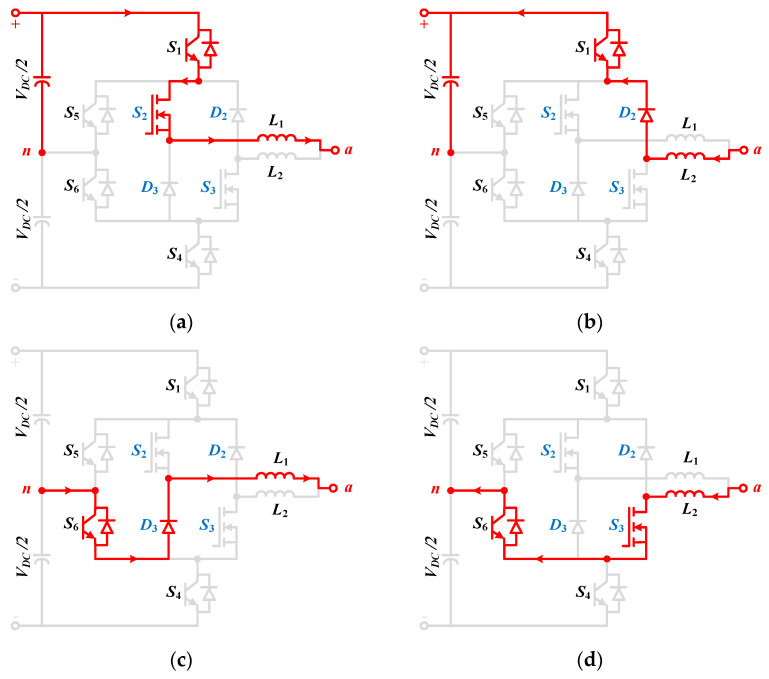
Switching paths of the hybrid ANPC inverter during positive half cycle: (**a**) mode 1 with *i_L_* > 0, (**b**) mode 1 with *i_L_* < 0, (**c**) mode 2 with *i_L_* > 0, and (**d**) mode 2 with *i_L_* < 0.

As illustrated in Figure 4, the LSPWM is employed for the output voltage generation. In addition, there are three voltage levels, namely, 0.5 *Vdc*, 0, and 0.5 *Vdc.* It can be observed from Figure 4 that the proposed inverter has four modes of operation. Mode 1 and mode 2 are for the first half cycle whereas mode 3 and mode 4 are for the negative half cycle. As both cycles have a symmetrical operation, only mode 1 and mode 2 are discussed in this paper as depicted in Figure 5.

### 2.3. Modes of Operation

As the operation is dependent on the directions of the load current, each mode has two cases. The detailed circuit operation for mode 1 and mode 2 is shown in Figure 4.

Mode 1: In this mode, the output will be a positive voltage. A two-output load current is possible in this case, as shown in Figure 5a,b for for *i_L_* > 0 and *i_L_* < 0, respectively. During this mode, the gate pulse is received only by *S*_1_, *S*_2_, and *S*_6_ switches while other switches remain OFF. In the case of *i_L_* > 0, the current will flow through the split inductor *L*_1_ because of the ON state of the switches *S*_1_ and *S*_2_. Similarly, when the load current direction is reversed, i.e., *i_L_* < 0, the current flows through another split portion of the inductor in *L*_2_. The inverter output voltage will be 0.5 *Vdc* in this mode irrespective of the load current direction, and it is depicted in Figure 4. Whether the load current is positive or negative, the current through the two split inductors, i.e., *L*_1_ or *L*_2_, will always be unidirectional. Therefore, unlike the split-NPC inverter that only can work for unity power factor because of one load direction current, the proposed hybrid ANPC inverter due to its two different load current direction can work on a wide range of power factors.

Mode 2: The operation of this mode is different from mode 1 because, here, the application of zero state, particularly *O_3_*, is performed. In other words, the state of the *S*_1_, *S*_4_, *S*_5_, and *S*_6_ switches will be the same as mode 1, however, the state of *S*_2_ and *S*_3_ will be changed so that zero output voltage can be obtained. It is evident that at the neutral point, the output voltage will be clamped. The identical current flow path can be seen in Figure 5c,d. In this case, when the load current is positive, the current will flow through switch *S*_6_ and the split portion of the inductor *L*_1_. Similarly, for a negative load current, the current path will be through switch *S*_3_ and other split portion *L*_2_ of the inductor. The analytical study for the inverter will be presented in the next section.

## 3. Performance Analysis, Results, and Discussions of Hybrid ANPC Inverter

By analyzing the switching states given in Table 3, the value of the output voltage can be derived. Since the output terminal has split inductors, the voltage depends on the variation in the inductor current. This ultimately means that if the rate of current change is large, the output voltage will see a decline because of the losses associated with the inductors. Therefore, the output voltages of the proposed inverter can be derived by using (1) and (2) for the positive half cycle and negative half cycle, respectively:

(1) $$V_{an}=0.5\times S_{2}V_{DC}-L_{1}\frac{di}{dt}$$

(2) $$V_{an}=L_{1}\frac{di}{dt}-0.5\times S_{3}V_{DC}$$

It is noticeable that as the current passes through *S*_2_ and *S*_3_, the current stress (*di*/*dt*) is declined considerably because of using Ga_2_O_3_ based switches. If (1) is utilized, then the rate of change of current through *S*_2_ for state transition from *O*_3_ state to *P* state can be calculated by:

(3) $$di=0.5\times S_{2}V_{DC}\times \frac{dt}{L_{1}}$$

Here, *dt* is denoted for the time interval for the *S*_2_ switch to transit from *O*_3_ state to *P* state. Typically, the turn ON (*t_on_*) time for each switch, including both Si and Ga_2_O_3_ switches will be comprised in this period. The nominal value of *dt* is acquired from the manufacturer’s datasheets for Si-based switches, whereas for Ga_2_O_3_, the information is obtained from [30]. The summary is demonstrated in Table 4. It is clear from expression (3) that the *di*/*dt* stress is inversely proportional to the value of the first split inductor (*L_1_*). This also pointed out the fact that as Ga_2_O_3_ switches have little *t_on_* time (approximately 28.6 ns), the split inductance (*L_1_*) value would be proportionally small to constrain the current stress of the ANPC inverter. Therefore, the voltage drop across *L_1_* would also be comparatively smaller than the DC-link voltage under steady-state operation. In addition to the reduced *di*/*dt* stress and voltage drop, under steady-state operating conditions, the inverter will experience reduced power loss across the inductor. Furthermore, as illustrated in Figure 1, the split inductors (*L_1_* and *L_2_*) are contributing to decoupling Ga_2_O_3_ switches *S_2_* from *D_2_* as well as *S_3_* from *D_3_*. The overshoots are significantly damped out because of this decoupling.

The common-mode voltage (CMV) of the hybrid ANPC inverter with 100 V DC-link can be calculated as follows:

(4) $$V_{an}=0.5\times 100-\frac{1\times 10^{-6}di}{28.6\times 10^{-9}}=50-34.96di.\;$$

The CMV of the conventional ANPC inverter with 100 V DC-link can be calculated as follows:

(5) $$V_{an}=100-\frac{1\times 10^{-6}di}{50\times 10^{-9}}=100-20di.\;$$

It can be observed that for a certain value of *di*, the CMV of hybrid ANPC is almost 64.96% less than the CMV of conventional ANPC.

### 3.1. Analysis of Shoot through Fault Protection

In the proposed inverter, the complimentary operation of *S*_2_ and *S*_3_ at high switching frequency may result in the false turn-on of the switches [30]. Since Miller capacitance is present in all switches, the stored charge in it can cause the false turn ON of *S*_3_. If both switches are in the ON state at the same time, the positive DC link voltage may become shorted in a positive half cycle of operation. The same thing is true for negative voltage during the negative half cycle. MOSFETs, in contrast to bipolar devices such as IGBTs, cannot endure overcurrent. Although shoot-through fault can happen in any switching device, since UWBG devices such as Ga_2_O_3_ switches are operating in this inverter at a very high frequency, they are more prone to this fault [31]. The issue is overcome by restricting the rate of the rising fault current using the split inductors. Hence, the proposed inverter configuration offers zero dead-band between *S_2_* and *S_3_*.

To observe the impact, the shoot-through fault is allowed to happen on purpose when transitioning from the zero state *O*_3_ to the state *P*. The fault current (*I_f_*) is allowed to pass through *S_2_* and can be determined by:

(6) $$I_{f}\left(t\right)=\frac{0.5\times V_{DC}}{R_{eq}+R_{1}+R_{2}}\left(1-e^{\frac{-t\left(R_{eq}+R_{1}+R_{2}\right)}{L_{eq}+L_{1}+L_{2}}}\right)$$

Here, *t* is the time interval when shooting through the fault is allowed to happen, the resistances of *L*_1_ and *L*_2_ are denoted by *R*_1_ and *R*_2,_ respectively, and, *R_eq_* and *L_eq_* are the equivalent resistance and inductance of the printed circuit board (PCB) path. *R_eq_* and *L_eq_* are required to calculate the maximum allowable time of shoot-through fault for a selected PCB.

The values of *R_eq_* and *L_eq_* are calculated to be 0.245 Ω and 187 nH, respectively, from the information given for PCB in [32,33]. Thus, the maximum allowable time is 21.06 ns for the selected design which is, in fact, lower than the turn OFF time of the Ga_2_O_3_ devices. In addition, the overcurrent limit for the design is 80 A. Therefore, before the switch *S*_2_ is turned off (with *t_off_* = 94 ns), the switch *S*_3_ will be turned on falsely and can cause device failure. This issue is resolved by allowing a shoot-through time which is almost twice the turn OFF time of the Ga_2_O_3_ devices by using 1 uH split inductors. The numerical calculations can be realized by:

For conventional ANPC with split inductors,

(7) $$I_{f}\left(t\right)=\frac{600}{0.245\;\Omega }\left(1-e^{\frac{-1004\;\mathrm{ns}\;\times 0.245\;\Omega }{2.187\;\mu H}}\right)=260.52\;A$$

For the proposed hybrid ANPC with split inductors,

(8) $$I_{f}\left(t\right)=\frac{600}{0.245\;\Omega }\left(1-e^{\frac{-188\;\mathrm{ns}\;\times 0.245\;\Omega }{2.187\;\mu H}}\right)=51.04\;A$$

**Figure 6:**
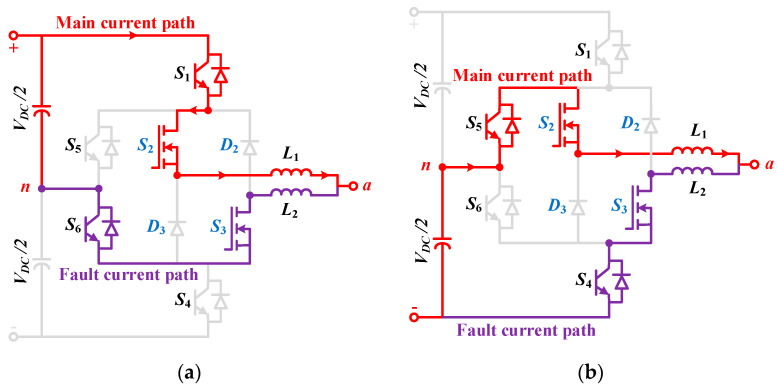
Shoot through fault current paths of the hybrid ANPC inverter during (**a**) state *O_3_* to state *P* for positive half cycle, (**b**) state *O*_2_ to state *N* for the negative half cycle.

**Figure 7:**
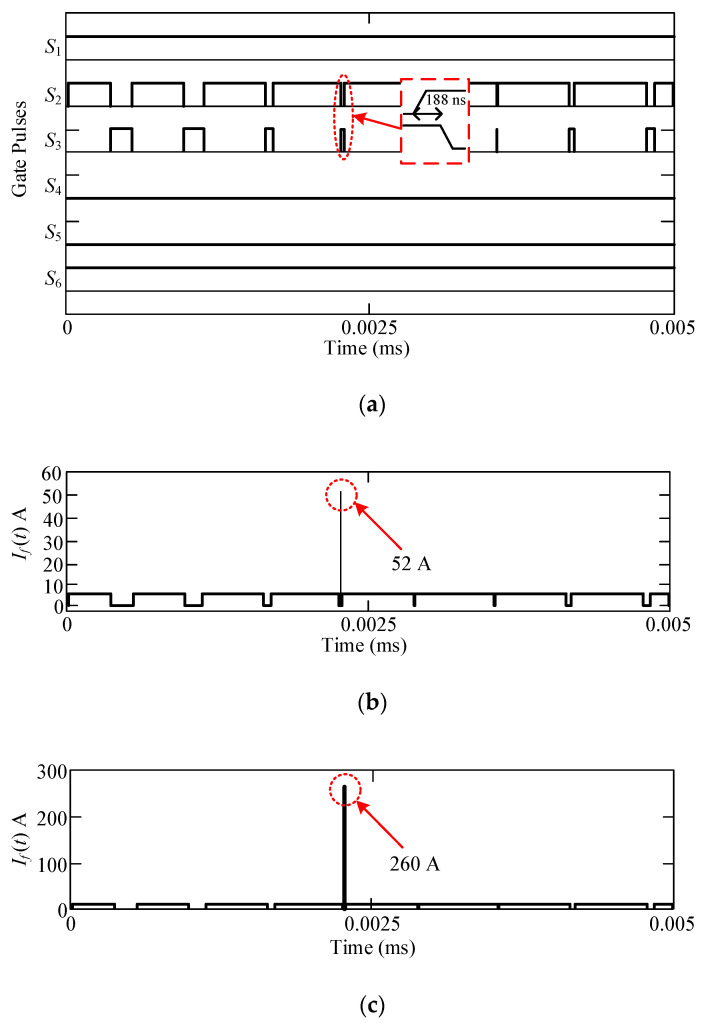
Shoot through fault current analysis: (**a**) gate pulses of *S_2_* and *S_3_* overlapping and causing shoot through fault, (**b**) hybrid ANPC, (**c**) conventional ANPC.

A simulation is conducted to determine the shoot through fault current of the proposed inverter by taking into consideration all the parasitic elements of the presented inverter circuit. Accordingly, the shoot-through fault’s current paths are illustrated for both the positive and negative half cycle in Figure 6a,b, respectively. The simulation results are shown in Figure 7, and it can be observed that they are almost similar to the calculated values. It can be observed that in the case of the proposed inverter, the fault current is within the limit. This validates the predominance of the UWBG device as well as the hybridization that has been utilized in this article. It is worth noting that the fault current can be reduced for the conventional ANPC by increasing the value of the split inductors. However, it will incur more inductor core losses into the system and will eventually reduce the inverter’s efficiency, making it radically unsuitable for industrial applications.

### 3.2. Analysis of Core Losses

The core losses of the proposed inverter are calculated in this section by considering the split inductors. The parameters which are considered for the proposed inverter’s inductor design are listed in Table 5. The permissible losses in the copper winding are computed for the chosen core, with the required product area, which is the product of the window area (*W_a_*) and the core area (*A_c_*):

(9) $$W_{a}\times A_{c}=\frac{LI_{max}I_{rms}}{K_{t}B_{max}j_{max}}$$

Here, *L* is one of the split inductors, *I_max_* is the maximum current flowing through the inductor, *I_rms_* is the rated RMS current, *K_t_* is the topological constant, *B_max_* is the maximum flux density, and *j_max_* is the maximum current density of the inductor. Although for complete accuracy the optimum loss for copper should be measured, the maximum permissible copper loss is calculated in this section because of the minimal difference between the accurate and approximate values, as well as for simplicity. Thus, the maximum allowable copper loss is used to measure the efficiency. The product area value obtained from (9) is used to determine the thermal resistance *R_th_* by utilizing the data from [33], assuming that the core temperature is increasing by 50 °C:

(10) $$R_{th}=17.45{\left(W_{a}\times A_{c}\right)}^{-0.509}+0.416\;℃/W$$

After the thermal resistance is calculated, this can lead to the measurement of maximum possible core loss (*P_Cu_*) for a particular temperature rise Δ*T*, and it can be determined by the following equation:

(11) $$P_{Cu}=\frac{\Delta T}{R_{th}}$$

**Figure 8:**
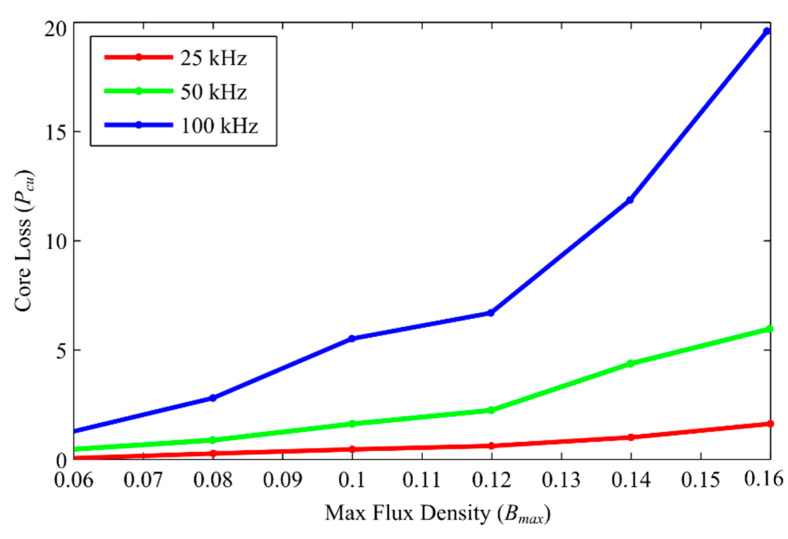
Core loss induced by the inductors under different switching frequencies.

The measurement of the copper winding loss can be performed for the split inductors by utilizing (9) to (11). Because of the minimal values of the product area, a large core size is selected for the practical design. The core losses for the selected material from Magnetics [34] are plotted using the values given in [33] in Figure 8 for the selected core volume.

### 3.3. Analysis of Switching Losses

Although it is already clear that the use of split inductors in the hybrid ANPC module is a major source of loss in steady-state operation, the inherent nature of the hybrid ANPC inverter is also responsible for the additional losses. The use of Ga_2_O_3_ switches *S*_2_ and *S*_3_ is a viable solution for this topology because these UWBG switches help to reduce the switching losses. Therefore, to quantify the improvement, it is essential to know how much loss is reduced after the addition of the UWBG switches.

**Figure 9:**
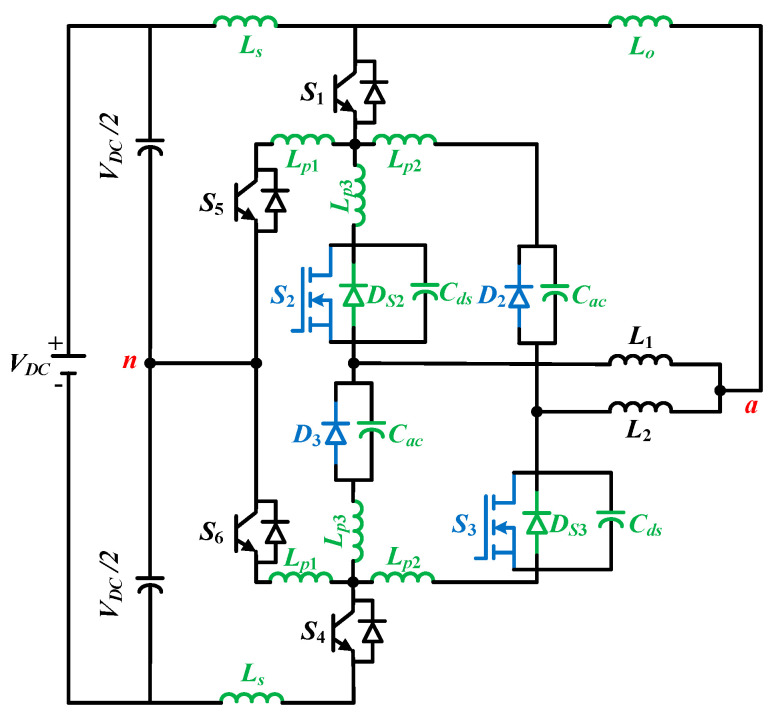
DPT circuit of the hybrid ANPC inverter with parasitic elements.

For switching loss measurement, double pulse testing (DPT) [30] is conducted. The DPT circuit used for the switching measurement is illustrated in Figure 9. The parasitic inductors in the PCB path are denoted by *L_p_*_1_, *L_p_*_2_, and *L_p_*_3_; the series inductor in the DC link is denoted by *L_s_*; and the output inductor is denoted by *L_o_*. Similarly, the drain to source capacitance of Ga_2_O_3_ switches and the anode–cathode capacitance of the Ga_2_O_3_ Schottky diode are indicated as *C_ds_* and *C_ac_*, respectively. The output inductance is measured following [30] while *L_p_*_1_, *L_p_*_2_, and *L_p_*_3_ are measured following [33]. All the calculated values are listed in Table 6.

Table 6 after putting these values in LT Spice, the simulation is conducted and switching transients are calculated.

**Figure 10:**
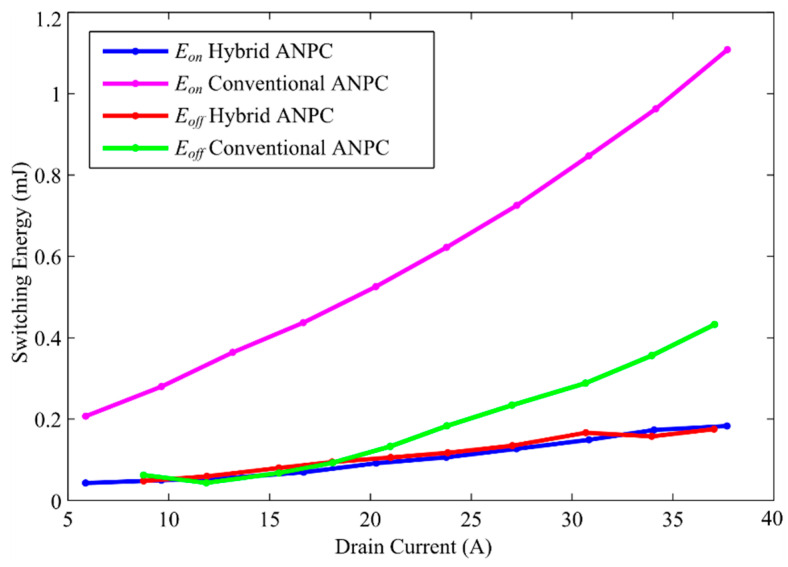
Characteristics curves highlighting the energies required for switches to turn ON and turn OFF with respect to the switching current for conventional ANPC and hybrid ANPC inverters.

**Figure 11:**
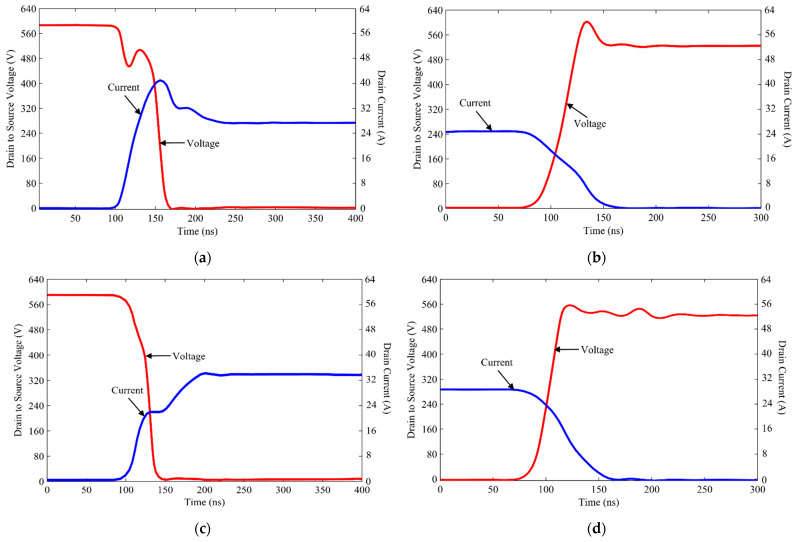
Switching curves of *S_2_* obtained from LTSpice: (**a**) switch turn ON for conventional ANPC, (**b**) switch turn OFF for conventional ANPC, (**c**) switch turn ON for hybrid ANPC, (**d**) switch turn OFF for hybrid ANPC.

The DPT test is performed repeatedly for different load currents and switching voltages to emulate practical scenarios. The data obtained from DPT are used to measure the energies required for the turning ON and turning OFF of the switches by using simulation, and they are referred to as *E_on_* and *E_off_*, respectively. Figure 10 illustrates the measured switching energies for both the conventional and the proposed inverter topologies. Though the energy consumption in the ideal switch should be zero, the semiconductor switches are hardly ideal, and thus, from these curves, it can be observed how switching energies rise when the load current increases. In addition, it is evident from these curves that the use of Ga_2_O_3_ switches has greatly contributed to reducing both the turn-on and turn-off switching energies. The simulated waveform shown in Figure 11 represents the minimization of switching losses with the utilization of Ga_2_O_3_ switches. It can be observed from Figure 11a that when *S*_2_ is turned on, the switching current has increased as soon as the gate pulse is applied. In other words, since conventional Si switches have a slow turn-on time, an overshoot current of 43 A is caused by *C_ac_* of *D*_3_. On the contrary, the Ga_2_O_3_ switches have a very fast turn-on time, which is why the overshoot current in this case significantly declined as shown in Figure 11c. This phenomenon also implies that due to the decreased overshoot, a faster decrease in switching voltage across the switch *S*_2_ in the case of the proposed inverter leads to decreased loss. In the case of turn-off, an almost similar event occurs in both case 1 and case 2, which are illustrated in Figure 11b,d, respectively. In this case, it can be observed that an overvoltage spike of almost 630 V is experienced by the conventional inverter compared to the 560 V spile of the hybrid.

ANPC inverter. This has resulted in higher turn OFF losses incurred by the conventional inverter. Although the margin of differences between the conventional inverter and the hybrid ANPC for turn OFF losses is very close, the overall switching losses of hybrid ANPCs are significantly lower because the turn ON losses are more dominant.

### 3.4. Analysis of Efficiency

The efficiencies of switching losses, conduction losses, and split-inductors losses are considered. The switching energies obtained from the DPT test are used for switching loss calculation. In case of switching loss, turn ON loss *P_on_* and turn OFF loss *P_off_* are determined by:

(12) $$P_{on}=f_{s}\times E_{on}$$

(13) $$P_{off}=f_{s}\times E_{off}$$

where the switching energies *E_on_* and *E_off_* can be determined by:

(14) $$E_{on}=I_{s}\times x_{on}$$

(15) $$E_{off}=I_{s}\times x_{off}$$

The equations for *x_on_* and *x_off_* can be mathematically expressed by:

(16) $$x_{on}=x_{1on}\times I_{s}{}^{2}+x_{2on}\times I_{s}+x_{3on}$$

(17) $$x_{off}=x_{1off}\times I_{s}{}^{2}+x_{2off}\times I_{s}+x_{3off}$$

Here, the constants *x*_1*on*_, *x*_2*on*_, *x*_3*on*_, …… are representative of the constants that are used for curve fitting shown in Figure 10. Additionally, the conduction losses are calculated using the manufacturer’s datasheet curves for different load currents in the case of conventional Si switches, whereas, for Ga_2_O_3_ switches, it has been obtained from the information provided in [35]. The expressions obtained from these curves are:

(18) $$P_{c}=x_{4}\times I_{s}{}^{2}+x_{5}\times I_{s}$$

where *x*_4_ and *x*_5_ are the constants for the curve fitting of Figure 10. Furthermore, the core losses from the split inductors are determined using the curves shown in Figure 8 and the information provided in [34].

**Figure 12:**
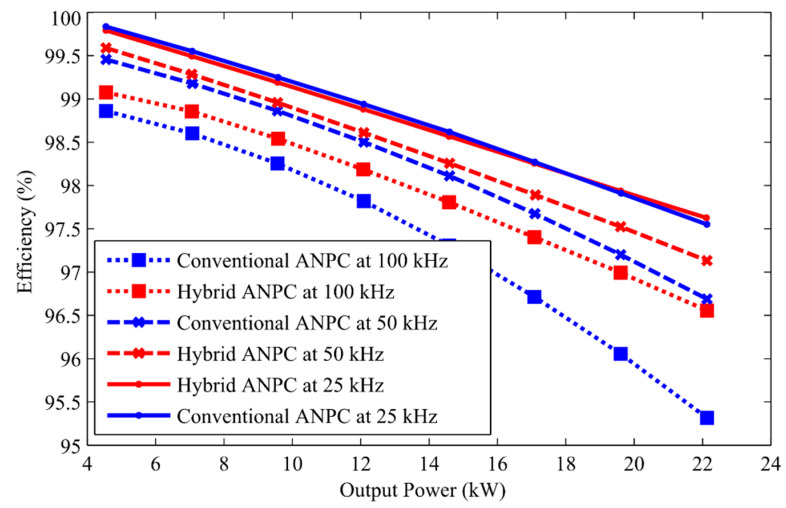
Efficiency comparison between conventional ANPC and hybrid ANPC under various switching frequencies.

In this paper, the losses of both conventional ANPCs as well as the proposed hybrid ANPC inverter are calculated considering different loads. In addition, three switching frequencies are considered to compare the loss behavior of the configurations, as shown in Figure 12. It can be validated from Figure 12 that because of using UWBG switches and due to reduced switching losses, the proposed inverter’s efficiency in all cases is much higher compared to the conventional Si-based ANPC inverter.

### 3.5. Analysis of High-Frequency Transient in Output Voltage

**Figure 13:**
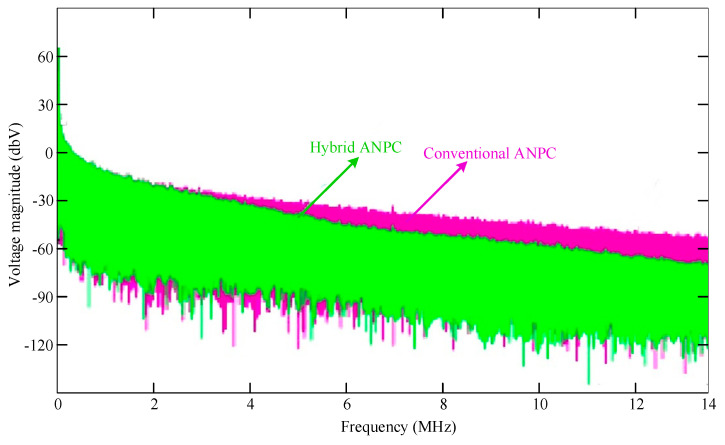
High-frequency voltage spectrum of the conventional ANPC and hybrid ANPC.

Along with the advantages of the conventional ANPC inverter, the proposed inverter can reduce high-frequency switching noise in the output voltage. This high-frequency noise primarily contributes to electromagnetic interference (EMI) issues and also has some impacts on the operation of the gate driver [24]. In addition, the incorporation of the two split inductors, i.e., *L*_1_ and *L*_2_, in the proposed inverter topology makes it possible to decrease the high-frequency transients considerably because of the filter of the transients by the inductances. Thus, the size of the electromagnetic compatibility (EMC) filter becomes significantly smaller. This statement can be validated by using (1) and (2). If any sudden change has occurred in the output voltage of the presented inverter, that impact will be damped by the inductance’s inherent capability to oppose any sudden change in current. The blocking voltage is tuned according to the values of the split inductor. For the proposed design, as the inductance value was 1 uH for the split inductor, the output voltages’ harmonic spectra can be illustrated for both conventional ANPCs and the proposed hybrid ANPC inverter through LT Spice simulation, as is illustrated in Figure 13. It can be seen that the final range of the high-frequency transient will be 5 to 15 MHz. This is due to the damped high-frequency voltage in this frequency range by the split-inductors. Thus, the added split inductors for the shoot-through protection also help to reduce the EMI filter size.

The cross-sectional area (*A*) of an EMI Filter for the hybrid ANPC with 100 kHz switching frequency can be determined by:

(19) $$A=\frac{2\pi rL}{\mu_{0}\mu_{r}N^{2}}=\frac{2\pi \times 0.1\times 0.5\times 10^{-6}}{1.2566\times 10^{-6}\times 6000\times 400}=1.04\times 10^{-7}m^{2}$$

Here, *r*, *μ_0_*, *μ_r_*, and *N* represent the toroid radius to centerline, the magnetic constant, the relative permeability of Mn–Zn ferrite, and the number of turns, respectively. Similarly, the cross-sectional area of the EMI filter for a conventional ANPC can be calculated as follows:

(20) $$A=\frac{2\pi rL}{\mu_{0}\mu_{r}N^{2}}=\frac{2\pi \times 0.1\times 20\times 50\times 10^{-9}}{1.2566\times 10^{-6}\times 6000\times 400}=2.08\times 10^{-7}m^{2}$$

**Figure 14:**
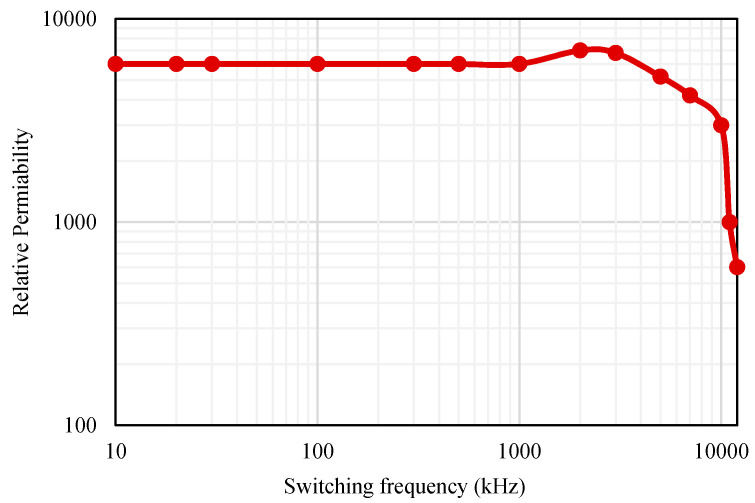
Relative permeability of Mn Zn ferrite under different switching frequencies.

Thus, it can be observed that the size of the EMI filter for the proposed ANPC inverters becomes halved compared to the conventional ANPC inverter due to the usage of split inductors. Furthermore, the relative permeability versus the switching frequency curve for Mn–Zn ferrite is shown in Figure 14. It is noticeable that with higher switching frequency, the relative permeability tends to decrease logarithmically. Therefore, the cross-sectional area of the EMI filter will increase with a higher switching frequency.

### 3.6. Analysis of Operation at Various Range of Power Factors

**Figure 15:**
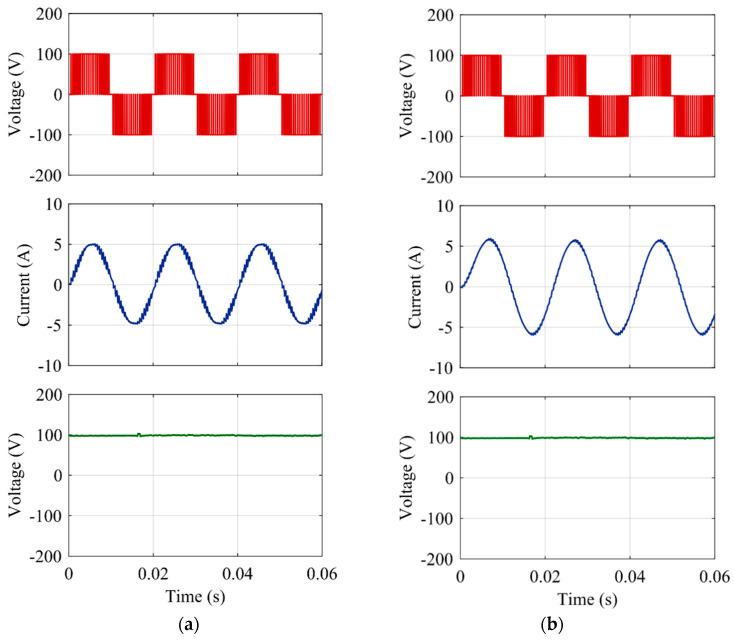
Simulation results for the hybrid ANPC inverter for the output voltage (*V_an_*), current (*I_an_*) and common-mode voltage (CMV) with (**a**) unity power factor, (**b**) non-unity power factor.

The MATLAB/Simulink version of the proposed hybrid ANPC inverter is developed in this section to validate that it can operate in various ranges of power factors. LTSpice simulation is not required in this case since this feature is embraced by the proposed inverter due to implementing the split-inductors-based design, and this feature is not associated with using UWBG switches. Thus, for operational simplicity, MATLAB Simulink along with ideal MOSFETs and IGBTs are used to develop the proposed inverter. The output voltage and current waveforms are obtained for the proposed topology using a 200 V DC link. Thus, a voltage of 100 V will come across each DC-link capacitor. The simulation tests are repeated with the loads with non-unity power factor. To show the applicability of the proposed converter compared to the existing topologies. The results show the non-distorted waveforms for voltage and currents. The results for output voltage *V_an_* and load current *I_an_* are shown in Figure 15. Furthermore, the voltage across one DC-link capacitor is also shown, which indicates the nature of the common-mode voltage (CMV). It can be observed that the CMV is always constant at 100 V and it does not contain any ripples of high frequency. Thus, the leakage-current-related issues can also be solved using this topology.

## 4. Conclusions

To sum up, this paper presents a three-level hybrid ANPC topology that includes Ga_2_O_3_-based MOSFET as well as Si-based IGBTs. This inverter has split inductors at the output, which are not only capable of protecting against the shoot-through fault but can also contribute to the reduced EMI in the output voltage. To maximize the efficiency of our converter, as well as to maximize the benefit of the Ga_2_O_3_ switches, both the modulation technique as well as four modes of operation are discussed in this paper. The efficiency of both the conventional ANPC and the proposed hybrid ANPC inverter is measured and compared through LT Spice and MATLAB simulations. It was observed that under various switching frequencies and output power, the minimum efficiency was 96.8%, whereas a 99.1% maximum efficiency was obtained by the proposed inverter. The employability of the proposed module is analyzed by taking into consideration the reduced overshoots in switching waveforms, higher efficiency, lower current, voltage stress, minimized shoot-through current, and EMI. Eliminating the dominating switching losses, especially turn-on losses, as well as the addition of UWBG switches, contributes to an increase in efficiency. In addition, to validate the inverter’s capability to supply reactive power, the module was operated under both various load conditions by changing the power factors. The simulation result acquired from the proposed module coincides with the theoretical results. The following is a list of the manuscript’s concluding statements:

- The proposed inverter incorporated UWBG-based Ga_2_O_3_ switches, which contributed to its enhanced efficiency and reduced switching losses.
- The Ga_2_O_3_ switches of the inverter make it a suitable candidate for high voltage, high temperature, and high switching operation.
- A maximum efficiency of 99.1% is obtained, making this inverter suitable for applications in grid-tied PV structures.
- The minimized EMI and fault current, because of the split-inductors-based design, allowed this inverter to be utilized in sophisticated industrial applications.

This study applies UWBG switches for ANPC inverters considering the technical pros and cons. Since the fabrication and production of UWBG semiconductors are still in their early phase industrially, experimental verification of the proposed inverter will be considered in the future. In the future, UWBG devices have great potential in the field of power electronics because of their superior characteristics over wide bandgap (WBG) and conventional semiconductors. Thus, researchers can utilize this opportunity to incorporate UWBG devices in other inverters/converter topologies and power electronic applications.

## Figures and Tables

**Table 1:** Parameters used in TCAD for analyzing conduction behavior of Ga_2_O_3_ switches.

Parameters	Values
Bandgap energy	4.8 eV
Effective density of states at 300 K	4.45 × 10^18^ cm^−3^
Electron affinity	4 eV
Electron mobility	118 cm^2^/Vs

**Table 2:** Parameters used in SPICE for analyzing switching behavior of Ga_2_O_3_ switches.

Parameters	Values
Channel length	2 μm
Channel width	4.7 × 10^6^ μm
Oxide thickness	20 nm
Electron mobility	118 cm^2^/Vs
Substrate doping	2 × 10^17^ cm^−3^
Zero-bias threshold voltage	−2.25 V
Transconductance	2.79 × 10^−6^ A/V^2^
Gate-drain capacitance	4.3 × 10^−11^ F/m

**Table 3:** Switching states for the proposed HANPC inverter.

States	Switches					
	*S*_1_	*S*_2_	*S*_3_	*S*_4_	*S*_5_	*S*_6_
*P*	1	1	0	0	0	1
*O*_1_	0	1	0	0	1	0
*O*_2_	0	1	0	1	1	0
*O*_3_	1	0	1	0	0	1
*O*_4_	0	0	1	0	0	1
*N*	0	0	1	1	1	0

**Table 4:** Switching parameters of Si and Ga_2_O_3_ switches.

Model	Switching Parameters			
	Rated Voltage (*V_r_*)	Rated Current (*I_r_*)	Turn on Time (*t_on_*)	Turn off Time (*t_off_*)
IGW15T120FKSA1 (Si IGBT)	1200 V	15 A	50 ns	502 ns
Ga_2_O_3_ switch	800 V	20 A	28.6 ns	94 ns
Ga_2_O_3_ Schottky diode	1700 V	25 A	-	-

**Table 5:** Parameters for designing the split inductors.

Parameters	Nomenclature	Values
Inductor	*L*_1_ = *L*_2_	1 μH
Maximum current	*I_max_*	42 A
RMS current	*I_rms_*	35 A
Topological constant	*K_t_*	0.3
Maximum flux density	B_max_	160 mT
Maximum current density	j_maz_	5 A/mm^2^
Product area	*W_a_* × *A_c_*	0.002 cm^4^

**Table 6:** Parameters for DPT testing.

Equipment	Nomenclature	Value
Inductors	*L_s_*	36.15 nH
	*L_p_*_1_	11.6 nH
	*L_p_*_2_	19.16 nH
	*L_p_*_3_	11.6 nH
	*L_o_*	1200 uH
Capacitors	*C_ds_*	171 pF
	*C_ac_*	80 pF
